# Development and application of a TaqMan-based real-time PCR method for the detection of the ASFV MGF505-7R gene

**DOI:** 10.3389/fvets.2023.1093733

**Published:** 2023-04-04

**Authors:** Chuanxiang Qi, Yongqiang Zhang, Zhenzhong Wang, Jinming Li, Yongxin Hu, Lin Li, Shengqiang Ge, Qinghua Wang, Yingli Wang, Xiaodong Wu, Zhiliang Wang

**Affiliations:** ^1^China Animal Health and Epidemiology Center, Qingdao, Shandong, China; ^2^MOE Joint International Research Laboratory of Animal Health and Food Safety, MOA Key Laboratory of Animal Bacteriology, College of Veterinary Medicine, Nanjing Agricultural University, Nanjing, China

**Keywords:** ASFV, MGF505 7R, qPCR, application, diagnosis

## Abstract

African swine fever virus (ASFV), the etiological agent of African swine fever (ASF), causes deadly hemorrhagic fever in domestic pigs. ASF's high mortality and morbidity have had disastrous effects on the world's swine industry. In recent years, the number of African swine virus strains has increased and presented new challenges for detecting classical ASFV-p72-based viruses. In this study, we observed that the ASFV MGF505-7R gene, a member of the multigene family that can enhance ASFV virulence and pathogenesis, has the potential to be a candidate for vaccine formulations. We also developed a real-time PCR assay based on the ASFV MGF505-7R gene and validated it in multiple aspects. The results indicated that the approach could detect standard plasmids with a sensitivity and a specificity of up to 1 × 10^1^ copies/μL. Moreover, the assay had no cross-reactions with other porcine viruses. In laboratory and clinical settings, the assay can detect ASFV-infected samples at an early stage (4 hpi) and show a consistency of 92.56% when compared with classical ASFV detection in clinically ASFV-infected materials. This study's results also indicated that the TaqMan-based quantitative real-time PCR assay we developed for detecting the ASFV MGF505-7R gene is both sensitive and specific. This assay can provide a quick and accurate method for detecting ASFV and has the potential to be used as an optional tool for screening and monitoring ASF outbreaks.

## 1. Introduction

African swine fever virus (ASFV), the etiologic agent of African swine fever (ASF), causes an acute, febrile, and highly contagious disease in domestic pigs and wild boars. Infected pigs exhibit symptoms such as high fever, loss of appetite, nasal discharges, and abortion, with a mortality rate close to 100% (1, 2). ASF can be spread through multiple modes, including a sylvatic cycle between swine and arthropod vectors, direct or indirect contact between the susceptible animal and infected pigs, and contaminated secretions or fomites (3). ASF is statutorily required by the World Organization for Animal Health (OIE) to be reported, and China also classifies ASF as a type I disease that needs to be controlled. Since the first ASF outbreak in China in 2018 (4, 5), nearly 1.2 million live pigs have been slaughtered, leading to significant socioeconomic consequences for the pig farming industry. To date, no effective vaccine or antiviral compound has been developed for ASF, while quarantine, depopulation, and sanitation strategies remain the common methods to curb the spread of the disease.

As the only member of the family *Asfarviridae*, ASFV has a large double-stranded DNA virus with a linear genome of 173–193 kb, encoding 151–167 open reading frames (ORFs) (6). To date, 24 different genotypes and eight serogroups have been identified based on the ASFV B646L (encoding the capsid protein p72) gene and the EP402R (encoding the serotype-specific protein CD2v) gene, respectively (7, 8). The ASFV multigene families (MGF) are characterized as genes present as repetitive sequences in the highly variable terminal genomic regions. MGF proteins vary widely between ASFV isolates due to frequent duplication, deletion, or inversion of MGF proteins (6). Given the great diversity of MGF, it will be of great interest and challenge to find a suitable target for ASFV diagnosis. At present, the ASFV p72 gene is commonly used for detecting ASFV due to its high conservation (9, 10), but as a late-transcription gene in ASFV (11), p72 shows low expression levels during the early stages of infection, making it difficult to accurately detect and measure viral loads, which may result in the misdiagnosis of some ASFV cases during the incubation period or early stages of infection. In this regard, we found MGF505-7R, a conserved gene from the MGF 505 family. MGF505-7R is located close to the end of the MGF family and thus shows little variation in most ASF strains. A study recently reported that MGF505-7R plays an important role in ASFV infection as it can be recognized by host CD8^+^ T cells and can potentially be used in vaccine formulations (12).

Additionally, MGF505-7R can enhance ASFV virulence and pathogenesis by inhibiting JAK1- and JAK2-mediated signaling (13) and suppressing the production of IL-1β and type I IFNs (14). The latest piece of research has shown that combinational deletions of MGF360-9L and MGF505-7R can attenuate highly virulent ASFV and provide protection against challenges (15). According to the above review, we believe that MGF505-7R is not only a conserved gene but also has a variety of potential functions that affect viral virulence, making it a promising candidate for detecting ASF.

Currently, there are various detection technologies available for ASFV, including virus isolation based on viral replication in susceptible cells (16, 17), ELISA based on the identification of ASFV antigens (18, 19), recombinase polymerase amplification (RPA) assay for virus gene detection (20, 21), CRISPR-Cas12a and fluorescence-based diagnosis system (22, 23), and loop-mediated isothermal amplification (LAMP) assay for the semi-quantitative analysis of ASFV (24). Despite these detection methods having advantages such as high accuracy, strong operability, and poor temperature requirements, several inconvenient requirements such as high costs, time-consuming nature, and low sensitivity may prevent them from becoming the best identification method (18, 25–27). Currently, the gold standard for ASFV diagnostics in laboratories is polymerase chain reaction (PCR), a viral genome detection technique based on nucleic acid marker amplification, and fluorescent quantitative PCR has become increasingly popular due to its high sensitivity, specificity, less time consumption, and accurate quantification. Fluorescence quantitative PCR mainly includes the SYBR Green method and the TaqMan probe method, and the latter has advantages in terms of specificity and accuracy and is highly applicable as it targets a sequence that is conserved in function, highly expressed during the early stages of infection, and functionally important, making it a valuable complementary target for ASFV diagnosis.

In this study, we developed a highly sensitive and specific TaqMan-based real-time quantitative PCR method for ASFV MGF 505-7R gene detection. The detection limit was low, at 1 × 10^1^ copies/μL, and exhibited good repeatability and specificity. In addition, our methods showed comparable results in laboratory and clinical samples compared to ASFV-p72-based qPCR diagnosis.

The development of the method produced a reliable candidate gene, increased the effectiveness of ASFV detection in the clinic, and may be further used to detect viral loads in various clinical samples from ASFV-infected pigs. The method also served as a technical tool for the pathogenicity study of ASFV, which helped researchers understand the virus better.

## 2. Materials and methods

### 2.1. Virus, plasmid, primers and probe

The African swine fever virus (ASFV) China_AnhuiXCGQ_2018 strain used in this study was preserved by the China Animal Health and Epidemiology Center (CAHEC), Shandong province, China (GenBank accession number MK128995.1). The full-length MGF505-7R gene (1584bp) in the p3xFLAG-CMV-7.1 vector, named p3xFLAG-MGF505-7R, was synthesized by Zhiyuan Biotechnology Co. Ltd. (Qingdao, Shandong, China). The recombinant vector was transformed into *E.coli* DH5α competent cells (CWBIO, Beijing, China) and extracted using the QIAGEN Plasmid Plus Maxi Kit. A pair of specific primers, MGF505-7R-F and MGF505-7R-R, and a TaqMan probe targeting MGF505-7R were designed with Oligo7 (Table 1). All primers and probes were also synthesized by Zhiyuan Biotechnology Co. Ltd. DNA and RNA concentrations were quantitated using a NanoDrop ND-1000 spectrophotometer and calculated in copy numbers.

**Table 1:** Primers and probes used in this study.

**Name**	**Primers (5'–3')**	**Size (bp)**
MGF505-7R-F	TAGGCAACAAATTCAAGGACT	131bp
MGF505-7R-R	CTTTTGTGACAACAGCAATGC	
MGF505-7R-probe	CGGAAGCTTGAGATTCTTACGTGGATGG	

### 2.2. Sequence analysis of ASFV MGF505-7R

The information of the MGF505-7R gene (QED90472.1, AYW34001.1, AKO62712.1, QBH90517.1, QBH90702.1, QDL88060.1, SPS73452.1, AJZ77061.1, QEY87835.1, AIY22221.1, AIY22380.1, CAN10378.1, CBW46695.1, QGM12819.1, CAN10129.1, AKO62711.1, CBH29131.1, AXB49776.1, AXB49605.1, AXB49949.1, AXB49433.1, AXB49259.1, and QED21587.1) from 39 different ASFV strains were collected from the GenBank and ASFVdb databases (28). Sequences were aligned using MEGA X software (29) and the clustalW method (30). Homolog analysis of ASFV MGF505-7R nucleotide sequences with multiple sequence alignment results was performed using the DNAMAN 7.0 tool (https://www.lynnon.com/dnaman.html).

### 2.3. Optimization of qPCR conditions

The qPCR assay was developed and validated using the CFX96Touch (Bio-Rad) and AceQ^®^ qPCR Probe Master Mix (Vazyme, China). The qPCR reaction system was determined as follows: AceQ qPCR Probe Master Mix (2X) of 10/μL, probe (10 pmol/μL) of 0.2 μL, upstream and downstream primers (10 pmol/μL) each of 0.4 μL, DNA of 1 μL, and used dd H_2_O to make up to the quantity of 20 μL. According to the designed primer Tm value, six different annealing temperatures were used with three replicates per group: 57, 58, 59, 60, 61, and 62°C, and 10^7^copies/μL p3xFLAG-MGF505-7R plasmid was used as a DNA template, and the optimum annealing temperature was selected based on the measurement results.

### 2.4. Standard curve

The DNA standard p3xFLAG-MGF505-7R was diluted 10-fold serially and amplified with the optimized qPCR system at a concentration of 1.0 × 10^7^-1.0 × 10^1^ copies/μL. A final standard curve was generated based on the CT value and the logarithm of the standard copy number.

### 2.5. Sensitivity

To determine the sensitivity of the assay, the p3xFLAG-MGF505-7R plasmid was diluted 10-fold serially with concentrations ranging from 1.0 × 10^7^-1.0 × 10^0^ copies/μL. Prepared standards were amplified with the optimized qPCR system to confirm the detection limit. At the same time, the conventional PCR was performed with the same DNA standard and primers. Amplifications were carried out in a 20 μL reaction system containing 10 μL 2 × Taq MasterMix (Vazyme, Nanjing, China), 1 μL of each primer, 2 μL of DNA, and 6 μL of RNase-free dd H_2_O. The thermal profile for the PCR was 95°C for 5 min, 35 cycles of amplification (30 s at 95°C, 30 s at 56°C, and 30 s at 72°C), and a final extension step at 72°C for 10 min. The resulting PCR products were analyzed by electrophoresis on an ethidium bromide-stained 1.5% agarose gel. We could compare the sensitivity between qPCR and PCR by observing their detection limits.

### 2.6. Specificity

The established real-time quantitative PCR method was used to detect common swine infectious viruses, including porcine pseudorabies virus (PRV), porcine reproductive and respiratory syndrome virus (PRRSV), porcine circovirus (PCV2), classical swine fever virus (CSFV), porcine parvovirus (PPV), and swine transmissible gastroenteritis virus (TGEV). A total of six viruses were used for testing the specificity of primers and probes. All viral nucleic acids and negative controls preserved by the laboratory were used as a template for a real-time fluorescence quantitative PCR reaction.

### 2.7. Repeatability

A repeatability test was carried out with p3xFLAG-MGF505-7R at concentrations of 1.0 × 10^7^-1.0 × 10^0^ copies/μL *via* an optimized protocol three times daily for 3 days to assess the accuracy of the method by calculating intra-assay and inter-assay standard deviations.

### 2.8. Laboratory samples testing

Porcine alveolar macrophages (PAMs) were used for laboratory sample preparation. To obtain PAMs, Bama pigs were raised for up to 4 weeks in the animal feeding room of the CAHEC. Cells were isolated from the lung lavage fluid (31) with only a slightly modified methodology, as previously described. Briefly, alveolar macrophages were obtained from 40-day-old crossbred piglets confirmed to be free of PCV, PPV, PRRSV, and ASFV by SYBR green-based qPCR and then maintained in RPMI-1640 medium with 10% fetal bovine serum (Hyclone) and 100 U/ml penicillin and 50 mg/ml streptomycin (Hyclone). PAM cells were maintained at 37°C with 5% CO_2_ overnight and then infected with 0.5 MOI China_AnhuiXCGQ_2018 at 4, 8, 16, 24, and 32 hpi. Cell samples were extracted for RNA using the FastPure Cell/Tissue Total RNA Isolation Kit V2 and then reverse transcribed into cDNA (Vazyme, Nanjing, China). Prepared templates were used for qPCR with the designed MGF505-7R probe and classic p72 probe (10). To reduce false-negative detections, the porcine housekeeping gene beta-actin (ACTB) was applied as an internal control. The results were analyzed with CFX Manager 3.0 (Bio-Rad) and GraphPad Prism 8 (GraphPad Software, San Diego, CA, USA).

### 2.9. Clinical samples testing

A total of 94 samples, including domestic pigs' blood, nose swabs, and lymph node tissues, were provided by the National Reference Laboratory for African Swine Fever in the China Animal Health and Epidemiology Center. Total DNA/RNA was extracted automatically from the samples using the Tianlong NP968-C Nucleic Acid Extractor with the GeneRotex96 program (TianLong Medtl, Hangzhou, China). ASFV was inactivated with 1% (w/v) of NaOH, as previously described in a BSL-3 laboratory (32), and viral DNA/RNA samples were prepared in a BLS-2 laboratory. Nucleic acids were extracted and then stored at −80°C for subsequent use. All these clinical samples were confirmed to be ASFV-positive with a p72-specific primer and probe. They used it for a qPCR assay of MGF505-7R. Samples with CT values below 40 were identified as ASFV-positive.

## 3. Results

### 3.1. Sequence analysis of ASFV MGF505-7R

**Figure 1:**
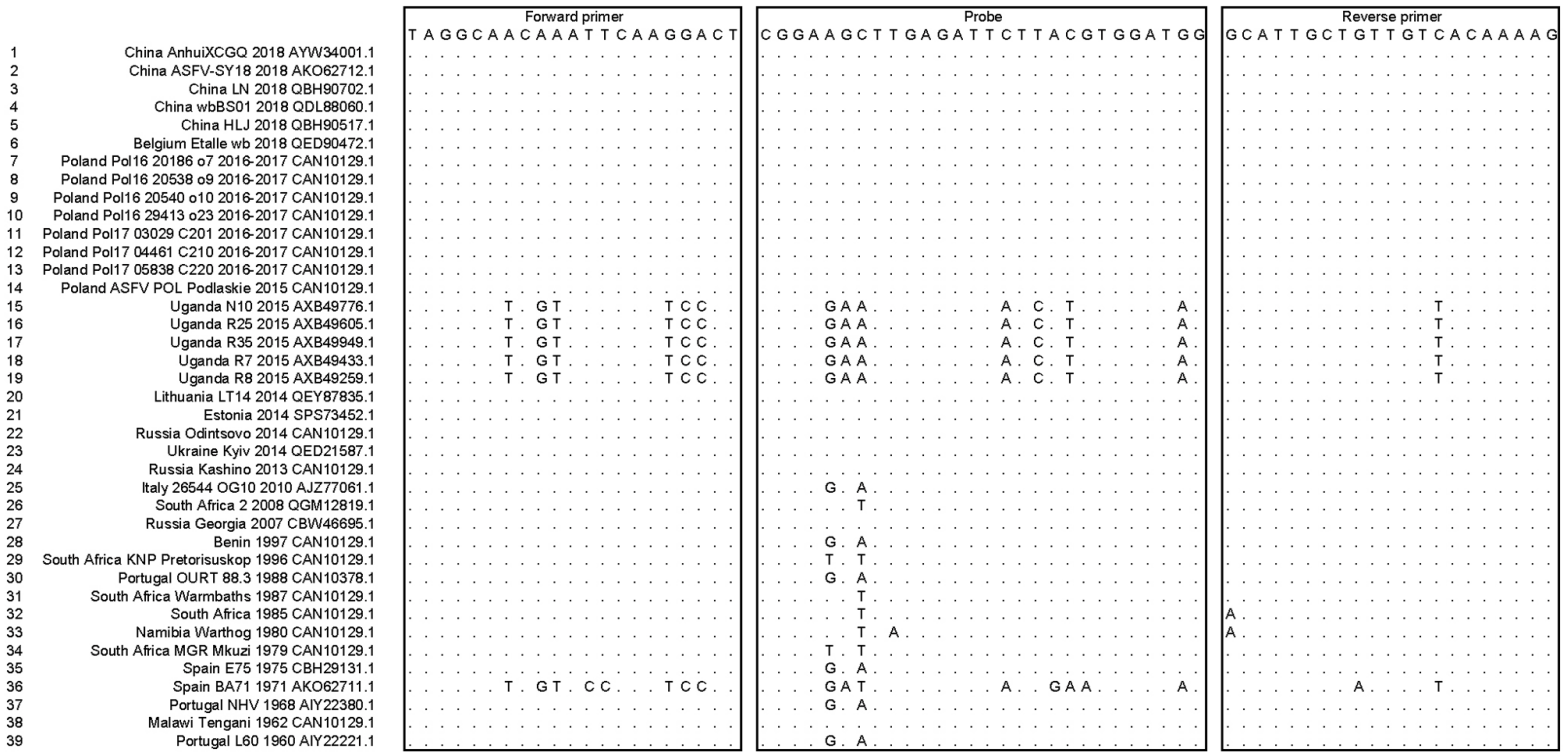
Alignment of the primers and probes targeted to the ASFV MGF505-7R genes among multiple ASFV strains. The sequences of the forward primers, probes, and reverse primers (used as an antisense oligonucleotide) were shown in boxes. Dots represented nucleotides identical to the primers and probes.

ASFV MGF505-7R nucleotide sequences from 39 different ASFV strains were involved in multiple sequence alignments, mainly including the ASFV genotype II strains epidemic in Asia and Europe in recent years. The homolog identification showed 96.68% identity, indicating that MGF505-7R is a relatively conserved gene. The sequences of the forward primers, probes, and reverse primers (used as an antisense oligonucleotide) targeting the ASFV MGF505-7R genes were also aligned, as shown in Figure 1. The mapping results of primers and probes with different strains were also listed. The data showed that the primers and probes we designed mapped well with most epidemic ASFV strains except five Ugandan strains (N10, R7, R8, R25, and R35) and the Spain_BA71 strain, which indicated that the designed assay might be able to detect ASFV MGF505-7R in Asian and European epidemic ASFV strains but not African ones.

### 3.2. Optimization of qPCR conditions

The p3xFLAG-MGF505-7R plasmid (10^7^copies/μL) was amplified by MGF505 7R probe-based qPCR with different annealing temperatures. CT values from different groups were compared. The results showed that, when the annealing temperature was 60°C, the minimum of the average CT values among the six annealing temperatures was 17.776, compared with other groups (57°C−18.185, 58°C−17.914, 59°C−17.890, 61°C−18.376 and 62°C−18.451). Therefore, we used an annealing temperature of 60°C for the real-time fluorescent quantitative PCR. They used a probe qPCR mix (2x) at a concentration of 10/μL, along with a probe (10 pmol/μL) and upstream and downstream primers (10 pmol/μL) at a concentration of 0.4μL and 0.6 μL, respectively. They also used 1μL of DNA and made up the final volume to 20 μL using dd H_2_O. The cycling conditions for real-time PCR reactions were 95°C for 1 min, followed by 40 cycles of 95°C for 5 s and 60°C for 60 s.

### 3.3. Standard curve

**Figure 2:**
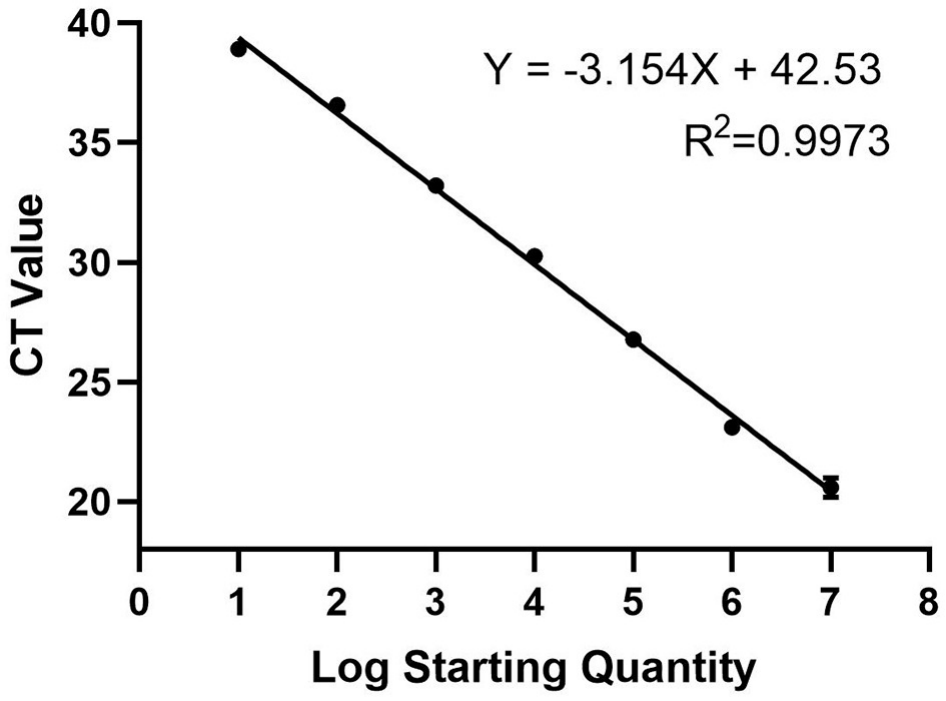
Standard curve for qPCR assay of MGF505-7R. A 10-fold serial dilution ranging from 1 × 10^7^ to 1 × 10^1^ copies/μL of the standard DNA was tested in the qPCR. Three replicates were tested for each dilution. The optimal standard formula is y = −3.154x + 42.53, and the correlation coefficient is 0.9973.

To construct a standard curve with the logarithm of the RNA copy number and the measured Ct value (Figure 2), serial p3xFLAG-MGF505-7R plasmid dilutions at the concentration of 1.0 × 10^7^ to 1.0 × 10^1^ copies/μL were prepared. Three replicates were tested for each dilution. The optimal curve was selected as the standard curve. The development of the MGF505-7R standard curve with the abscissa as the logarithm of copy number and the ordinate as CT value revealed that the correlation coefficient (R2) was 0.9986, the slope was −3.301, and the intercept was 39.416. The standard formula is *y* = −3.301x + 39.416 and R^2^ = 0.9986.

### 3.4. Sensitivity

**Figure 3:**
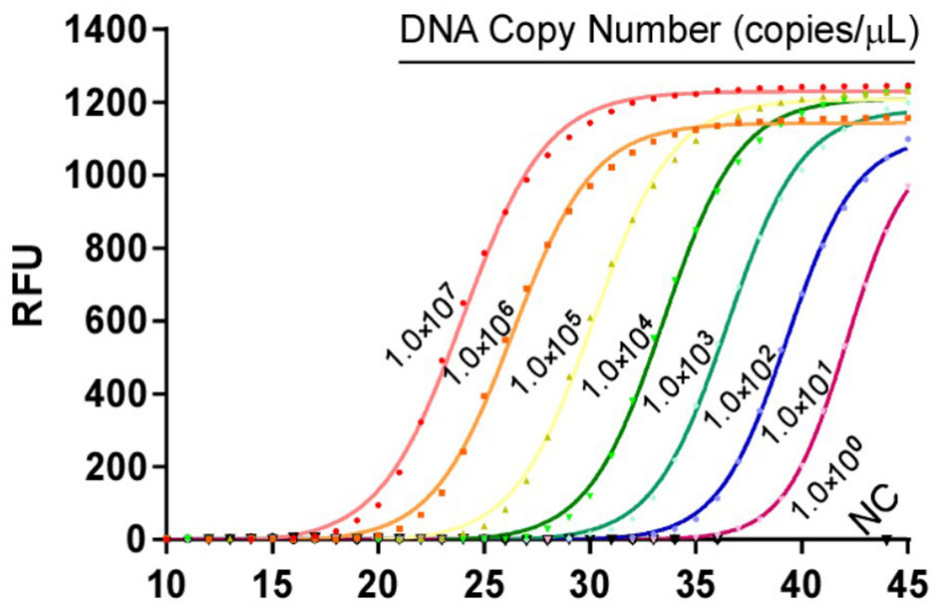
Sensitivity tests for qPCR assays based on the MGF505-7R probe. 10-fold serial dilutions of the DNA standard were used to perform the qPCR to obtain the standard curve of the assay. The lowest copy number that could be determined was up to 10 copies/μL.

**Figure 4:**
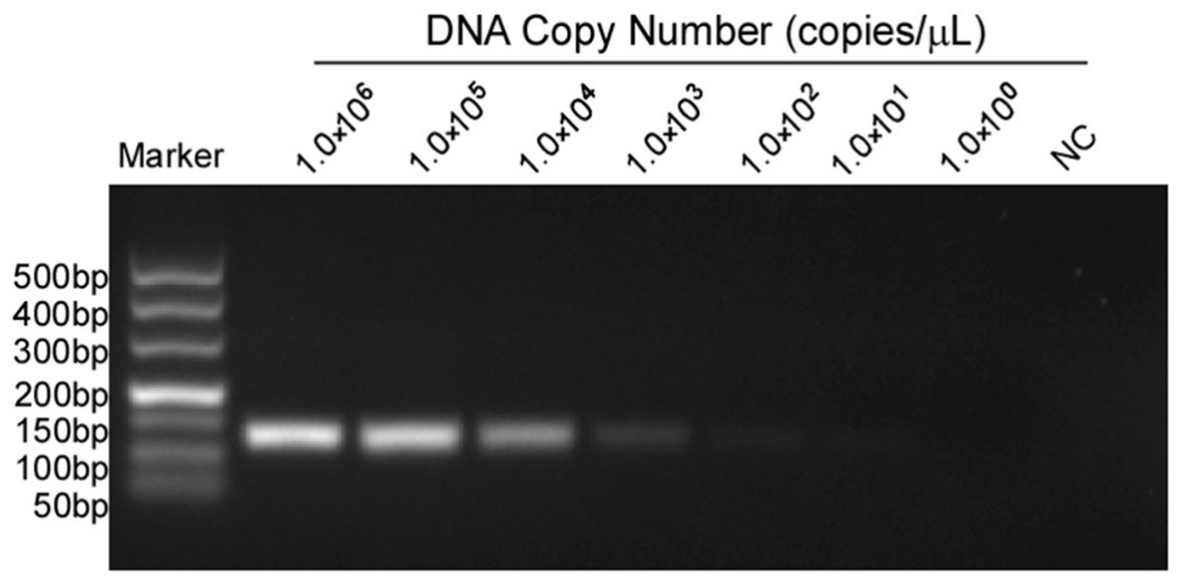
Sensitivity tests for conventional PCR of MGF505-7R. Lanes 1 to 7 were the templates, with concentrations ranging from 1 × 10^6^ to 1 × 10^0^ copies/μL. Lane 8 was a negative control. The lowest copy number that could be determined was up to 1.0 × 10^2^ copies/μL.

The 10-fold gradient dilution of the standard plasmid was simultaneously detected by the established qPCR and conventional RT-PCR. The minimum detection template concentrations of the established qPCR and conventional RT-PCR were 10 copies/μL and 1.0 × 10^2^copies/μL, respectively (Figures 3, 4), indicating that the qPCR was more sensitive than conventional RT-PCR.

### 3.5. Specificity

**Figure 5:**
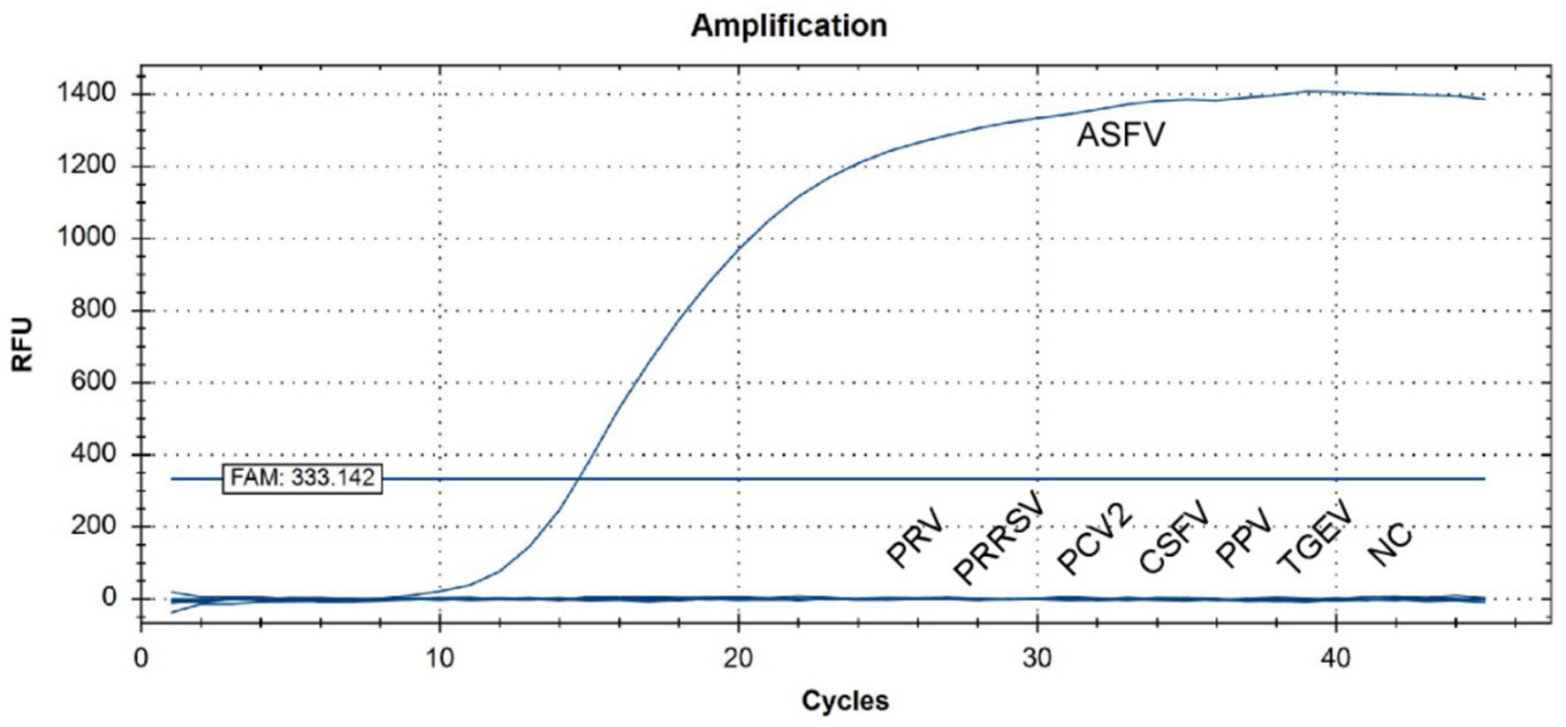
QPCR amplification results of the specificity assay. Standard nucleic acid materials, including porcine pseudorabies virus (PRV), porcine reproductive and respiratory syndrome virus (PRRSV), porcine circovirus (PCV2), classical swine fever virus (CSFV), porcine parvovirus (PPV), and swine transmissible gastroenteritis virus (TGEV), were tested together with ASFV nucleic acid. An extra negative control (NC) was set at the same time. qPCR data showed that only ASFV was detected with a positive fluorescence signal, and no other positive signal was observed in the other viruses or the negative control.

MGF505-7R from the Shandong province was selected for amplifications in a qPCR assay, and strong fluorescent signals were obtained from reactions. However, for other pig viruses such as PRV, PRRSV, CSFV, PPV, PCV2, and TGEV, data showed that the assay gave no signal amplification (Figure 5). Therefore, there is a significant distinction between ASFV and other viruses when comparing the signal strength at different levels.

### 3.6. Repeatability

The intra-assay repeatability was assessed by testing 10-fold serial dilutions of the standard plasmid ranging from 1 × 10^7^ to 1 × 10^1^ copies/μL in three replicates. The same standard plasmid was analyzed in triplicate on three different days to determine the inter-assay repeatability. As a result, intra-assay standard deviations (SD) ranged from 0.11 to 0.21, and inter-assay standard deviations (SD) ranged from 0.12 to 0.22. The coefficient of variation (CV) values of intra-assay and inter-assay were 0.06% to 1.06% and 0.55 to 0.81%, respectively (Table 2). The results showed that the experiment was repeatable.

**Table 2:** The repeatability of the developed qPCR.

**Standard Copies/μL**	**Intra-assay repeatability of CT-value**			**Inter-assay repeatability of CT-value**		
	**Mean**	**SD**	**CV (%)**	**Mean**	**SD**	**CV (%)**
1.0 × 10^7^	19.82	0.15	0.75	20.59	0.04	0.2
1.0 × 10^6^	23.01	0.07	0.28	23.1	0.03	0.15
1.0 × 10^5^	26.13	0.1	0.4	26.78	0.02	0.06
1.0 × 10^4^	29.65	0.14	0.48	30.25	0.02	0.06
1.0 × 10^3^	33.05	0.2	0.62	33.04	0.24	0.72
1.0 × 10^2^	35.7	0.08	0.22	36.23	0.2	0.56
1.0 × 10^1^	38.42	0.24	0.63	38.91	0.4	1.02

### 3.7. Laboratory samples and clinical samples

**Figure 6:**
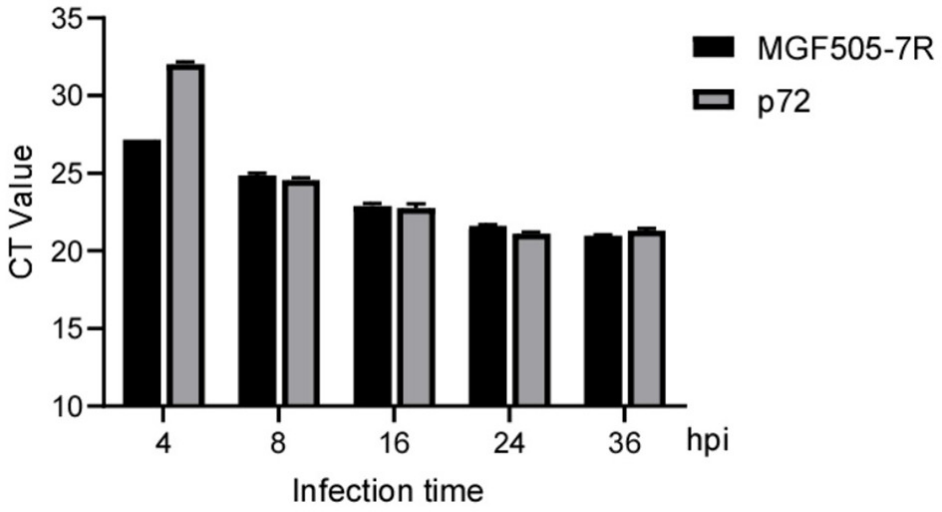
Comparison of amplification results between MGF505-7R and p72 in PAM cells at different time points. PAM cells were infected with ASFV (MOI = 0.5), and samples were collected by RNA extraction at indicated time points for qPCR detection with the MGF505-7R probe or the ASFV-p72 probe. The figure shows the CT value with three duplicates at each time point.

**Figure 7:**
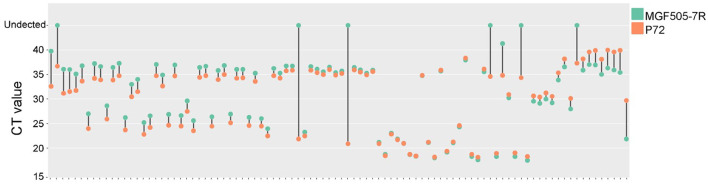
Comparison of amplification results between MGF505-7R and P72 in clinical samples. The dumbbell diagram shows the CT value of each clinical sample based on the MGF505-7R probe (blue) and p72 probe (red), respectively.

For laboratory-made ASFV-infected samples, MGF505-7R probe-based qPCR showed a lower CT value than the p72 probe at 4 hpi. For the remaining 8, 16, 24, and 36 hpi samples, the two groups obtained similar results (Figure 6). For clinical samples, the classical ASFV p72-based detection method showed 100% positives, and 87 out of 94 (92.56%) samples were determined to be MGF505-7R positive by our assay. The data showed that seven samples were diagnosed as ASFV MGF505-7R negative, including six samples showing no signal and one with a CT value higher than 40. Further comparison found that 32 samples showed lower CT values in the MGF505-7R-based group than in the p72-based group. The remaining 55 samples showed higher CT values (Figure 7). The assay based on MGF505-7R showed results close to a classical test method from the preliminary detection results of clinical samples.

## 4. Discussion

ASF is a highly contagious disease that causes acute hemorrhagic fever in domestic and wild boars, with a fatality rate of up to 100%. After being effectively eradicated from areas outside of Africa and Sardinia in the late 1990s, the “second wave” of ASF that spread cross-continentally to Europe and Asia in 2007 became a major concern for the global pig industry (3). Unfortunately, the cross-regional transport of pigs and pork products, the lack of good farming practices and biosecurity, and the mobility of people and vehicles all contribute to the disease's future spread, which has expensive socioeconomic effects on the affected countries. Given that there is currently no viable vaccine or antiviral agent treatment available, early detection and diagnosis of ASFV in various settings, including farms and slaughterhouses, are urgently required to manage the disease, and are considered the primary method for management.

ASFV is difficult to diagnose, especially outside of a diagnostics laboratory, because it exhibits symptoms similar to those of classical swine fever (CSF) and a number of other swine diseases (33). Therefore, several diagnostic techniques, including ELISA, RPA, and LAMP, have been developed and formally certified for diagnosing ASF. Because of its precision and effectiveness, qPCR stands out among these techniques and is currently widely used despite the limitations of laboratory technology (34). In recent years, classical detection methods have gradually satisfied the increasing number of ASFV strains; therefore, developing candidate detection genes can be of great importance.

In our study, we described a particular real-time quantitative PCR assay based on TaqMan for detecting ASFV MGF505-7R. According to the abovementioned data, qPCR has a detection limit as low as 10 copies/μL, making it 100 times more sensitive than a conventional PCR experiment. Additionally, no signals were detected in the test with any other virus. The intra-assay and inter-assay variances ranged from 0.06 to 1.02%, demonstrating the repeatability of the approach. In conclusion, compared to the traditional RT-PCR method, this study's TaqMan-based real-time qPCR method was quick, sensitive, and specific. The assay was run over a broad dynamic range with little intra- and inter-assay variation, and it revealed no cross-reactivity with many other pig-origin viruses. In an *in vitro* study, MGF505-7R was detected with a lower CT value early in infected PAMs, suggesting that this method can also be applied to laboratory studies. In the results of clinical samples, this assay also showed a similar effect to the standard method for ASFV diagnosis.

The ASFV-p72 protein is highly conserved among different viral strains and has stable antigenicity, which makes it the most common marker for detecting ASFV. In this experiment, we found that the CT value of MGF505-7R became stable and credible as early as 4 hpi, while the expression of p72 tends to be significantly stable at 8 hpi. By comparing the mRNA levels of MGF505-7R and p72 at each stage of ASFV infection, we found similar results to viral gene transcriptome analysis (11), indicating that MGF505-7R has the potential to be a promising candidate for ASFV detection.

Although our method has shown some good characteristics, there is still room for improvement. We observed that the primers and probe in our assay could cover most epidemic ASFV strains from Asia and Europe but not those from Africa (Uganda strains). Therefore, in the future, additional probes and primers may need to be considered for the MGF505-7R gene to supplement this method for better detection.

The development of MGF505-7R-based TaqMan real-time PCR for ASFV diagnosis has shown superior results in identifying the presence of ASFV in the early infection stage compared to the classic method *in vitro*. This method also provides a quantifiable research tool for studying the function of the MGF505-7R gene and investigating the epidemiology of ASFV infections in pigs, which could also facilitate research into the epidemiology of ASFV infections in other animals.

## Data availability statement

The original contributions presented in the study are included in the article/supplementary material, further inquiries can be directed to the corresponding authors.

## Ethics statement

The animal study was reviewed and approved by the Animal Welfare Committee of the China Animal Health and Epidemiology Center, and written informed consent was obtained from the owners for the participation of their animals in this study.

## Author contributions

CQ, YZ, ZheW, ZhiW, and XW conceived and designed the experiments. CQ, YZ, ZheW, and JL performed the experiments. CQ and YZ analyzed the data and wrote the manuscript. YH, LL, QW, and YW contributed reagents/materials/analysis tools. All authors contributed to the article and approved the submitted version.
